# Antibacterial Monoclonal Antibodies Do Not Disrupt the Intestinal Microbiome or Its Function

**DOI:** 10.1128/AAC.02347-19

**Published:** 2020-04-21

**Authors:** Omari Jones-Nelson, Andrey Tovchigrechko, Matthew S. Glover, Fiona Fernandes, Udaya Rangaswamy, Hui Liu, David E. Tabor, Jonathan Boyd, Paul Warrener, Jose Martinez, Jamese J. Hilliard, C. Ken Stover, Wen Yu, Gina DAngelo, Sonja Hess, Taylor S. Cohen, Bret R. Sellman

**Affiliations:** a Department of Microbial Sciences, AstraZeneca, Gaithersburg, Maryland, USA; b Antibody Discovery and Protein Expression, AstraZeneca, Gaithersburg, Maryland, USA; c Data Science & AI, AstraZeneca, Gaithersburg, Maryland, USA; d Translational Sciences, AstraZeneca, Gaithersburg, Maryland, USA; e Statistical Sciences, AstraZeneca, Gaithersburg, Maryland, USA

**Keywords:** microbiome, antibiotics, monoclonal antibodies

## Abstract

Antibiotics revolutionized the treatment of infectious diseases; however, it is now clear that broad-spectrum antibiotics alter the composition and function of the host’s microbiome. The microbiome plays a key role in human health, and its perturbation is increasingly recognized as contributing to many human diseases. Widespread broad-spectrum antibiotic use has also resulted in the emergence of multidrug-resistant pathogens, spurring the development of pathogen-specific strategies such as monoclonal antibodies (MAbs) to combat bacterial infection. Not only are pathogen-specific approaches not expected to induce resistance in nontargeted bacteria, but they are hypothesized to have minimal impact on the gut microbiome. Here, we compare the effects of antibiotics, pathogen-specific MAbs, and their controls (saline or control IgG [c-IgG]) on the gut microbiome of 7-week-old, female, C57BL/6 mice. The magnitude of change in taxonomic abundance, bacterial diversity, and bacterial metabolites, including short-chain fatty acids (SCFA) and bile acids in the fecal pellets from mice treated with pathogen-specific MAbs, was no different from that with animals treated with saline or an IgG control. Conversely, dramatic changes were observed in the relative abundance, as well as alpha and beta diversity, of the fecal microbiome and bacterial metabolites in the feces of all antibiotic-treated mice. Taken together, these results indicate that pathogen-specific MAbs do not alter the fecal microbiome like broad-spectrum antibiotics and may represent a safer, more-targeted approach to antibacterial therapy.

## Abstract (precis)

Antibiotics revolutionized the treatment of infectious diseases; however, it is now clear that broad-spectrum antibiotics alter the composition and function of the host’s microbiome. The microbiome plays a key role in human health, and its perturbation is increasingly recognized as contributing to many human diseases. Widespread broad-spectrum antibiotic use has also resulted in the emergence of multidrug-resistant pathogens, spurring the development of pathogen-specific strategies such as monoclonal antibodies (MAbs) to combat bacterial infection.

## INTRODUCTION

Until recently, microbiology research has focused primarily on the bacterial pathogens responsible for infection or chronic disease, ignoring the 100 trillion microbes making up the microbiome that occupies our skin and mucosal surfaces (1). In the last decade, the microbiome has become increasingly recognized as an integral part of human health, immunity, and the response to certain therapeutics. In particular, the gastrointestinal tract microbiome has been reported to play a key role in maintaining health and homeostasis. In fact, disruption of the microbiome, or dysbiosis, has been linked to diseases such as diabetes, obesity, and asthma (2, 3). Although many factors such as diet, travel, and where we live can affect the bacterial composition of our microbiota, it is unlikely that anything directly impacts its composition as much as broad-spectrum antibiotic therapy does (4).

Antibiotics have saved countless lives and have enabled modern medical treatments, including organ transplants, joint replacement surgeries, and immunosuppressive cancer therapies (5). However, broad-spectrum antibiotic use has expanded beyond treating serious bacterial infections into agriculture to promote livestock growth and are often prescribed to treat colds and upper respiratory tract infections that are likely caused by a virus (6, 7). Such widespread antibiotic use has fueled the current resistance epidemic, which some fear will lead us back into a preantibiotic era (8). Additionally, recent studies have demonstrated both an acute and sustained impact of broad-spectrum antibiotics on the composition and function of the human microbiome which can adversely affect human health (9). For example, patients exposed to broad-spectrum antibiotics are at increased risk of Salmonella enterica serovar Typhimurium-induced colitis or recurrent Clostridioides difficile infections (10, 11). Also, children administered antibiotics during the first year of life were found to exhibit an increased risk for developing asthma and childhood obesity (12, 13). Antibiotic use by patients suffering from intestinal diseases such as inflammatory bowel disease (IBD) has been correlated with decreased bacterial diversity in the gut and increased intestinal inflammation (14, 15), highlighting a need for alternatives to broad-spectrum antibiotics.

Antibiotic-mediated dysbiosis not only alters the bacterial population resulting in reduced bacterial diversity in the gut, but it can also alter levels of key metabolites, such as short-chain fatty acids (SCFAs), or the conversion of primary bile acids into secondary bile acids (16, 17). SCFAs are produced by fermenting gut bacteria and play an integral role regulating the intestinal epithelial barrier through tight junction proteins (TJP), influence immunity by driving regulatory T cell (Treg) differentiation, and affect satiety and insulin production (18–21). Constituents of a healthy microbiome also convert primary bile acids produced by the liver into secondary bile acids, which impact host inflammation, immunity, and lipid and glucose metabolism (22, 23). Dysbiosis of the gut microbiome following antibiotic exposure has been shown to impact bile acid metabolism and, consequently, affect immune tone (24, 25). It is therefore clear that alterations in gut bacterial populations can lead to changes in key metabolic pathways that impact human health and well being.

This greater understanding of the impact antibiotic-mediated dysbiosis has on human health along with the ongoing antibiotic resistance epidemic has led to the exploration of new methods to prevent or treat bacterial infections. Monoclonal antibodies (MAbs) are an attractive option due to their target specificity, long half-life, and ability to synergize with the host’s immune response (26–28). Here, we studied the gut microbiome of specific-pathogen-free (SPF) mice following treatment with antibacterial MAbs or broad-spectrum antibiotics to determine if pathogen-specific MAbs alter the host microbiome or its metabolic products.

## RESULTS

### Antibacterial MAbs do not change the bacterial density in feces.

**FIG 1:**
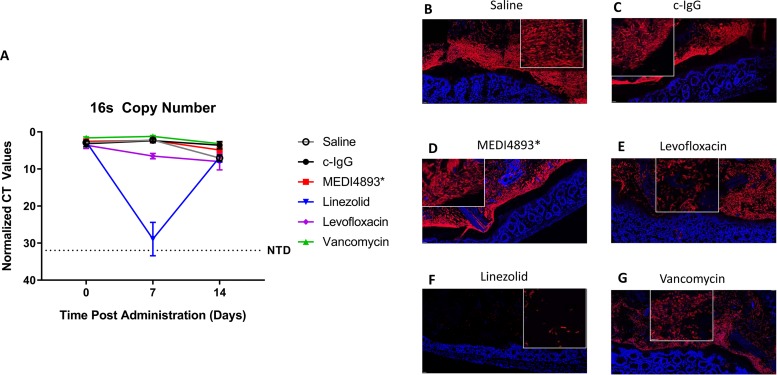
Antibacterial MAbs do not change the microbial concentration in feces. (A) The relative concentrations of bacteria, as measured by 16S qPCR, in fecal samples of mice at day 0 (prior to treatment) and at 7 and 14 days posttreatment with saline, c-IgG, MEDI4893*, levofloxacin, linezolid, or vancomycin. (B to G) Segments of mouse colon with fecal pellet at 7 days posttreatment fixed in Carnoy’s solution and stained with red 16S probe for bacteria in the feces and lumen; blue indicates DAPI staining the nuclei of the epithelium. Inset shows bacteria at increased magnification. All data are representative of at least two independent experiments, *n *= 5/group.

We hypothesized that unlike antibiotics (Abx), pathogen-specific MAbs would not alter the number or composition of bacteria in the intestinal microbiome. Specific-pathogen-free (SPF) mice were treated with a single dose of antibacterial MAb on day 0 or with a human-equivalent dose of broad-spectrum antibiotics for 5 days. A single dose of MAb was given because the half-life of human IgG in mice is 7 to 10 days, and MAbs such as MEDI4893 and MEDI3902 are administered as a single dose in human clinical trials (29, 30). Fecal samples were collected on days 0, 7, and 14 from mice treated with a clinical candidate MAb targeting Staphylococcus aureus (MEDI4893*) or human-equivalent doses of antibiotics used to treat S. aureus infections (vancomycin [VAN], levofloxacin [LVX], and linezolid [LZD]). Relative concentrations of bacteria, as measured by 16S rRNA gene quantitative PCR (qPCR), in fecal samples from mice treated with control IgG (c-IgG), MEDI4893*, or vancomycin were unchanged relative to the concentrations in saline controls at all time points. Conversely, the number of fecal bacteria was significantly reduced on day 7 in mice treated with levofloxacin or linezolid (*P* = 0.0083 and 0.0126, respectively) compared with saline-treated controls but was not significantly different than saline-treated controls on day 14 (Fig. 1A). Sections of colon containing a fecal pellet were removed, and fluorescent *in situ* hybridization (FISH) with a 16S rRNA gene probe was used to image bacteria within the colon. Bacterial density along the colonic epithelium was similar in mice treated with c-IgG, MED4893*, and saline. Antibiotic treatments noticeably affected the bacteria in the colon at day 7 posttreatment, with levofloxacin and linezolid reducing the bacterial burden and vancomycin eliminating all rod-shaped bacteria (Fig. 1B to G). Treatment of mice with anti-Pseudomonas aeruginosa MAb MEDI3902 or anti-Klebsiella pneumoniae MAb KPE33 also did not alter the overall bacterial content, while the antibiotic meropenem significantly (*P* = 0.0003) reduced bacterial load (see Fig. S1A and B in the supplemental material). These data demonstrate that unlike most of the antibiotics tested, the pathogen-specific MAbs have a negligible effect on the overall size of the bacterial population present in murine feces.

### Antibacterial MAbs do not change the bacterial composition of feces.

The 16S rRNA gene V4 region was sequenced to determine the effect the different antibacterial therapies had on the overall microbial community structure and the abundances of individual taxa.

**FIG 2:**
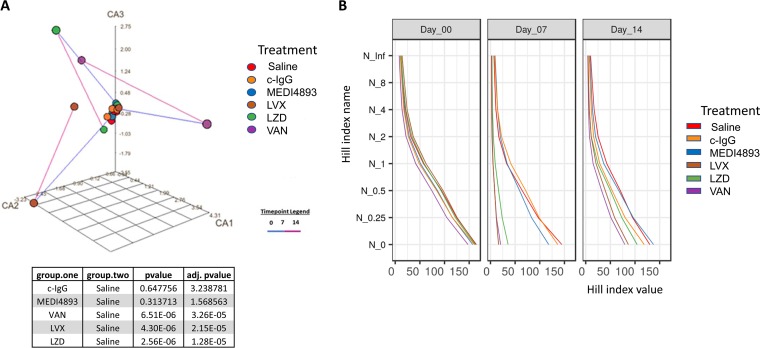
Antibacterial MAbs do not change the overall microbial composition of feces. (A) Correspondence analysis (CA) plot of microbiota in fecal samples treated with saline, c-IgG, MEDI4893*, levofloxacin (LVX), linezolid (LZD), or vancomycin (VAN). Each point represents the geometric median of the genus taxonomic profiles within each treatment group at days 0 (prior to treatment), 7, and 14. The time point legend indicates the period of collection, with the blue line extending from time points 0 to 7 and the purple line extending from time points 7 to 14. adj., adjusted. (B) Plots of the abundance-based diversity indices (Hill numbers), also known as the effective numbers of species. The vertical axis corresponds to different values of the order parameter *q* that defines the Hill index N*_q_* (66). The horizontal axis shows the respective index values. Taxonomic profiles of individual observations were aggregated into geometric medians for each combination of treatment and time of sample collection. Each such aggregated community is represented by one Hill index series shown as a line. One community is more diverse than the other if the values in the entire Hill series are higher for the first community (i.e., the lines for the two communities do not cross). All data are representative of at least two independent experiments, *n* = 5/group.

Correspondence analysis (CA) plots of the Bray-Curtis dissimilarity between the geometric medians of the taxonomic profiles of different treatment groups (Fig. 2A and S2A and B) revealed dramatic changes in the overall taxonomic composition on day 7 in antibiotic-treated groups. That was followed by a shift closer to the original state on day 14 in the levofloxacin and linezolid groups, while the vancomycin group remained in a more perturbed state. In contrast, treatment with pathogen-specific MAbs MEDI4893*, MEDI3902, or KPE33 resulted in much smaller compositional shifts during the course of treatment, comparable to those of the saline or c-IgG controls.

In the richness and alpha-diversity analyses, we observed that the administration of the broad-spectrum antibiotics levofloxacin, linezolid, vancomycin, ciprofloxacin, or meropenem dramatically reduced the Hill numbers in the treated samples on day 7, whereas the Hill numbers from the antibacterial MAb-treated samples remained similar to those of the controls (Fig. 2B and S2C and D).

**FIG 3:**
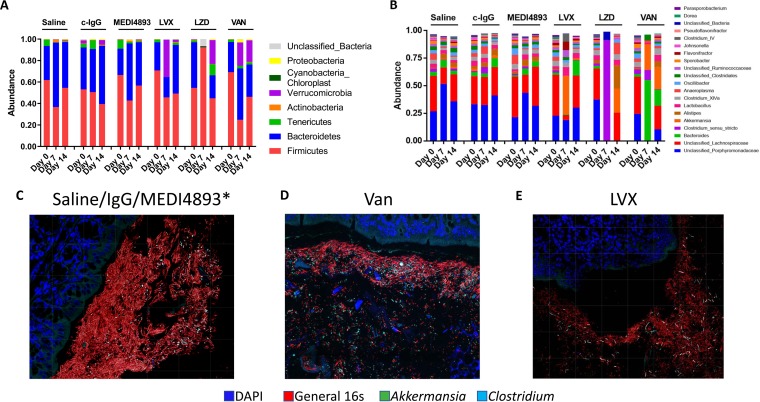
Antibacterial MAbs do not change bacterial taxonomic abundances in feces. (A) Relative abundances of major bacterial phyla of mice treated with saline, c-IgG, MEDI4893*, levofloxacin (LVX), linezolid (LZD), or vancomycin (VAN) at day 0 (prior to treatment) and days 7 and 14. (B) The relative abundance of major bacterial genera of treated mice at days 0 (prior to treatment), 7, and 14. (C to E) Segments of mouse colon with fecal pellet 7 days posttreatment stained with red 16S probe for bacteria, green for *Akkermansia* spp., and cyan for *Clostridium sensu stricto*; blue indicates DAPI staining. All data are representative of at least two independent experiments, *n* = 5/group.

Prior to treatment, the fecal microbiota of naive mice was mostly composed of bacteria from the *Bacteroidetes* and *Firmicutes* phyla (Fig. 3A). *Porphyromonadaceae* (∼25%) and *Lachnospiraceae* (∼25%) had the highest abundances at the family level (Fig. 3B). Similar taxonomic composition was observed in day 7 and 14 fecal samples from mice treated with MEDI4893*, c-IgG, or saline. Levofloxacin most strongly affected *Lachnospiraceae* on day 7, but that treatment group returned to the taxonomic distribution observed in the saline-treated controls by day 14. Linezolid resulted in a taxonomic profile dominated by Clostridium *sensu stricto* on day 7 and remained perturbed on day 14. Vancomycin reduced the relative abundance of the Gram-positive *Firmicutes* phylum on day 7, corresponding to a reduction in *Porphyromonadaceae* and *Lachnospiraceae* families and an increase in the relative abundance of members of the Akkermansia genus. The taxonomic profile of that treatment group remained perturbed on day 14. FISH was used to probe for specific taxa that were observed to be high in abundance within the fecal microbiota. Relatively high abundances of *Akkermansia* and Bacteroides spp. were observed in the colon sections collected from the mice treated with levofloxacin and vancomycin, respectively. These relative abundance proportions were not observed in the mice treated with saline, c-IgG, or MEDI4893* (Fig. 3C to E). Similar outcomes were observed with MAbs MEDI3902 and KPE33, which behaved like the saline controls, whereas additional antibiotics, ciprofloxacin and meropenem, were disrupted in the microbiota, particularly on day 7 (Fig. S3A to D).

### Antibacterial MAbs do not affect the levels of SCFAs or bile acids.

**FIG 4:**
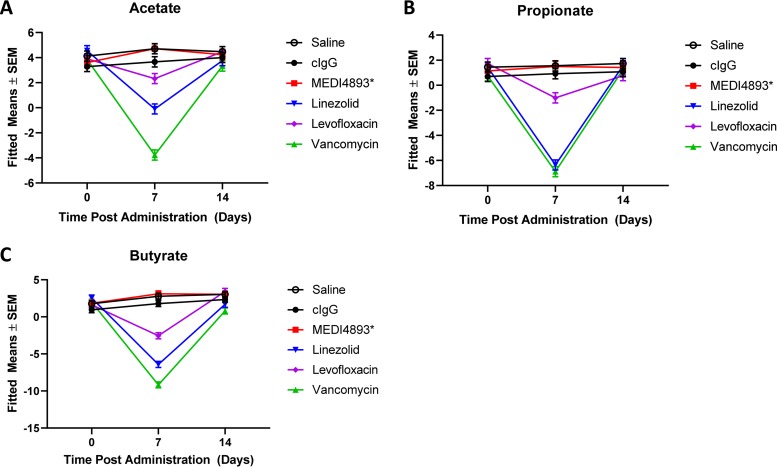
Antibacterial MAbs do not affect the levels of SCFAs. (A to C) Relative abundances of acetate (A), propionate (B), and butyrate (C) in fecal samples of mice treated with saline, MEDI4893*, levofloxacin, linezolid, or vancomycin at day 0 and days 7 and 14 posttreatment. All data are representative of at least two independent experiments, *n* = 5/group. SEM, standard error of the mean.

SCFAs, notably, acetate, propionate, or butyrate, can be produced or influence several bacteria within the *Firmicutes* phylum (*Lachnospiraceae* and *Lactobacillus*) and the *Bacteroidetes* phylum (*Porphyromonadaceae* and Alistipes spp.) (31–34). Decreases in the relative abundances of acetate, propionate, and butyrate were observed in fecal samples from the mice treated with vancomycin, linezolid, or levofloxacin on day 7 in comparison to the controls. Consistent with pathogen-specific MAbs not affecting the fecal microbiome, the SCFA levels in the MEDI4893*-treated samples remained similar to those of the saline control (Fig. 4A to C).

**FIG 5:**
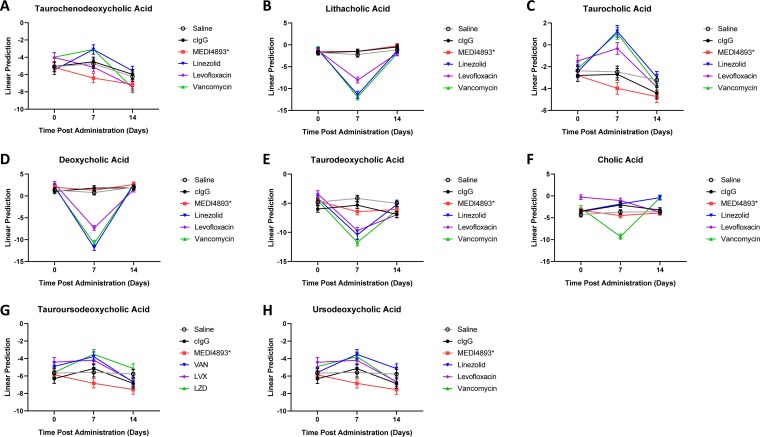
Antibacterial MAbs do not affect bile acid metabolism. (A to H) Relative abundances of taurochenodeoxycholic acid (A), lithocholic acid (B), taurine-conjugated lithocholic acid (C), deoxycholic acid (D), taurodeoxycholic acid (E), cholic acid (F), tauroursodeoxycholic acid (G), and ursodeoxycholic acid (H) in fecal samples of mice treated with saline, MEDI4893*, levofloxacin, linezolid, or vancomycin at day 0 and days 7 and 14 posttreatment. All data are representative of at least two independent experiments, *n* = 5/group.

Recent studies acknowledged the role of bile acids in health and disease (35). Changes were observed in the conversion of primary bile acids (BAs) into secondary BAs in mice treated with vancomycin, linezolid, or levofloxacin. Antibiotic-treated samples exhibited reduced the conversion of taurochenodeoxycholic acid (TCDCA) into the secondary BAs lithocholic acid (LA) or taurine-conjugated lithocholic acid (TLCA) and taurocholic acid (TCA) into deoxycholic acid (DCA) or taurodeoxycholic acid (TDCA) in comparison to the controls. In contrast, conversion of the primary BAs into secondary BAs in the MAb-treated samples remained similar to that of the controls (Fig. 5A to F).

## DISCUSSION

Antibiotics save countless lives; however, recent evidence demonstrates that antibiotics can have major off-target effects on the gut microbiome. The human gut microbiome is an “external organ” that is integral to human health and should be considered as part of the risk assessment process for new drugs. Antibiotic perturbation of a healthy microbiome coupled with the emergence and expansion of antibiotic-resistant bacterial pathogens have necessitated the development of novel antibacterial strategies that limit some of these adverse effects. Pathogen-specific MAbs have emerged as one such strategy due to their precision targeting and low risk for off-target effects. Antibodies have unique properties which enable them to promote pathogen clearance through multiple mechanisms, including virulence factor neutralization, inhibition of bacterial clumping and biofilm formation, and engagement of the immune system through Fc-dependent interactions (36). MAbs targeting the major human pathogens S. aureus, P. aeruginosa, and K. pneumoniae have a minimal effect on the gut microbiome compared with standard-of-care antibiotics.

Alterations to the microbiome have been linked to numerous diseases, as dysbiosis can influence key functions of the microbiome (3). Bacteria in the gut act not just as a physical barrier against colonization by pathogenic organisms but also by metabolizing small molecules which then circulate throughout the host, influencing numerous host processes. Our data demonstrate that pathogen-specific MAbs do not reduce the absolute abundance, diversity, or taxonomic composition of the gut microbiome compared to those with the effects of antibiotic treatment. Such effects observed in the antibiotic treatment groups are indicative of a reduced bacterial burden in the gut and diminished capacity to prevent infection by opportunistic pathogens (37–40).

Additionally, metabolic functions of the microbiome were not altered in the presence of MAbs as they were with antibiotics. The host relies on bacterial metabolism not only to process food but also to break down metabolites such as SCFAs and bile acids (41, 42). Altered processing of either SCFAs or bile acids due to restructuring of the microbiome has been linked to metabolic diseases such as diabetes and nonalcoholic fatty liver disease (NAFLD) (42–46). Functional consequences of microbiome disruption are not limited to metabolic disease. Autoimmune disease, graft-versus-host disease, allergies, and inflammatory bowel syndrome have all been linked to microbiome dysbiosis (47–50). Furthermore, both the development of different types of cancer and the response rates to current immune-targeted cancer therapies have been linked to the structure and function of the microbiome (51–54). Many of these microbiome-related diseases are associated with bacterial infection, and we postulate that antibiotic treatment of concurrent infections could exacerbate the underlying disease by modifying the microbiome. Due to their minimal impact on the microbiome, pathogen-specific MAbs would avoid such a complication.

The symbiotic relationship between our immune system and the bacteria which colonize us is increasingly recognized as having influence over a wide array of biological processes. This occurs through microbe-dependent production of SCFAs, bile acids, and other metabolites. We demonstrate that unlike standard-of-care antibiotics, pathogen-specific approaches for the treatment of infectious diseases do not alter the composition or function of the gut microbiome. MAb-based approaches have the potential to work synergistically with the host, reducing the duration and/or amount of overall antibiotic use, resulting in reduced impacts on human health.

## MATERIALS AND METHODS

### Reagents.

MAbs were diluted and prepared fresh daily from refrigerated or frozen stocks into sterile phosphate buffer saline (PBS; pH 7.2). The anti-S. aureus alpha toxin MAb MEDI4893*, anti-P. aeruginosa MAb MEDI3902, and anti-K. pneumoniae MAbs KPE33 and KPN42 were previously described (26, 55, 56). Isotype control human IgG1 (c-IgG) was included as a control. Analytical-grade vancomycin, linezolid, levofloxacin, ciprofloxacin, and meropenem were corrected for potency and prepared fresh daily.

### Mice.

All animal studies were approved by the AstraZeneca Institutional Animal Care and Use Committee and were conducted in an Association for Accreditation and Assessment Laboratory Animal Care (AAALAC)-accredited facility in compliance with U.S. regulations governing the housing and use of animals. Specific-pathogen-free 7- to 8-week-old female C57BL/6J mice (The Jackson Laboratory) were ear tagged, randomized, and housed in sterile cages with autoclaved mouse chow (LabDiet 5K52) and water. At least 5 mice per group were used in each experiment.

### Study design.

Animals were injected intraperitoneally (i.p.) with either a single 0.5-ml MAb dose (15 mg/kg of body weight) or administered human-equivalent doses of vancomycin (100 mg/kg twice a day [BID]), linezolid (60 mg/kg BID), ciprofloxacin (10 mg/kg BID), meropenem (66.7 mg/kg BID), or levofloxacin (235 mg/kg once a day [QID]) in 0.2 ml via subcutaneous (s.c.) injection for 5 consecutive days. Animals treated with PBS vehicle (BID) were included in each study as a control. Two fecal pellets (∼40 mg total) were collected from each animal on days 0 (prior to treatment), 7 (after a 36-h washout period), and 14 and immediately processed for DNA extraction (see below). A single fecal pellet was collected from the same animals and immediately stored at –80°C for SCFA and bile acid analyses. In select experiments, an additional cohort of mice from each group was euthanized by CO_2_ asphyxiation on day 7 for intestinal fluorescent *in situ* hybridization (FISH) staining.

### DNA extraction and quantification.

DNA was extracted from mouse feces using the PowerSoil DNA isolation kit (Mo Bio, West Carlsbad CA, USA), according to the manufacturer’s protocol, and DNA concentrations were determined using a NanoDrop 1000 spectrophotometer (Thermo Fisher Scientific). Purified DNA was stored at −80°C until use. The universal qPCR 16S rRNA primers U16SRT-F (5′-ACTCCTACGGGAGGCAGCGT-3′) and U16SRT-R (5′-TATTACCGCGGCTGCTGCTGGC-3′) were used to quantify total bacterial 16S rRNA genes from the purified fecal DNA (19). qPCRs were performed using 2 μl of template DNA from 2 fecal pellets per mouse, 2 pmol each primer, 3 μl of deionized water, and 5 μl of Sybr green master mix (Promega). The cycling conditions were as follows: 50°C for 2 min, 95°C for 2 min, 95°C for 15 s, and 60°C for 1 min, followed by a dissociation stage for 40 cycles. Differences in threshold cycle (*C_T_*) values were compared following normalization of DNA input in each qPCR.

### Library preparation and sequencing.

16S rRNA gene PCR was carried out as described previously (57). Briefly, V4 16S rRNA gene libraries were constructed using AccuPrime *Taq* high-fidelity DNA polymerase (Invitrogen). Library cleanup and normalization were performed using the SequalPrep normalization plate (96-well) kit (Invitrogen). Libraries were then pooled, and the final concentration of the library was determined using a Qubit fluorometer (Thermo Fisher Scientific). Libraries were mixed with PhiX control v3 (Illumina) and denatured using fresh 0.04 N NaOH. Pooled libraries were sequenced on an Illumina MiSeq instrument using a 2 × 250-bp MiSeq reagent kit v2.

### Sequence analysis.

Sequences were trimmed from adaptors using BBTools (58) and processed into amplicon sequence variants (ASVs) using the R package Dada2 (59), with taxonomy assigned from the RDP (60) training set v.14, as maintained by the Dada2 repository.

### Fluorescence *in situ* hybridization of colonic microbiome.

To visualize the colonic microbiome, colon sections containing a fecal pellet were processed as previously described (61). Briefly, the tissue was fixed in Carnoy’s solution for 2 weeks and paraffin embedded. Slides were made from 5-μm slices, which were deparaffinized in xylene (20 min at room temperature), followed by a 5-min incubation in 99.5% ethanol. The tissue was then incubated overnight at 50°C in hybridization solution (20 mM Tris-HCl, 0.9 M NaCl, 0.1% SDS, 25% formamide) containing the following FISH probes: 16S rRNA, GCTGCCTCCCGTAGGAGT; *Akkermansia* spp., GGTTCCCCTCCATTAC; *Clostridium sensu stricto*, GCCGTGGCTTCCTCCTY; *Porphyromonas* spp., CTCGTTATGGCACTTAAGCCGA; and *Bacteroides* spp., CGCAATCGGAGTTCTTCGTGATATC (62). Nuclei were stained with 4′,6-diamidino-2-phenylindole (DAPI), and the slides were washed 3× with PBS and coverslipped with ProLong Gold mountant (Invitrogen). Images were collected using a Zeiss LSM 880 Airyscan or Leica SP8 microscope. Following acquisition, images were deconvolved using Airyscan processing or Leica Lightning deconvolution software.

### Metabolite extraction from fecal samples.

Fecal samples were stored at –80°C prior to metabolite extraction. Metabolite extraction was performed with 50:50 acetonitrile-water. The amount of extraction solvent added to each sample was normalized to sample mass at a ratio of 10 μl extraction solvent to 1 mg sample. After the addition of extraction solvent, samples were manually homogenized and mixed at 2,000 rpm for 15 min at room temperature. Samples were centrifuged for 10 min at 18,000 × *g* and 4°C. One hundred microliters of supernatant was transferred to new tubes and centrifuged for 10 min at 18,000 × *g* and 4°C. Twenty microliters of supernatant was transferred to new tubes for derivatization of SCFAs as described below. Ten microliters of unlabeled metabolite extract was combined with 90 μl bile acid internal standard composed of a mixture of 15 bile acids at 1 μM in 50:50 MeOH-H_2_O. The bile acid internal standard mixture contains the following deuterated bile acids: lithocholic acid-d_4_, chenodeoxycholic acid-d_4_, deoxycholic acid-d_4_, ursodeoxycholic acid-d_4_, cholic acid-d_4_, glycolithocholic acid-d_4_, glycochenodeoxycholic acid-d_4_, glycodeoxycholic acid-d_4_, glycoursodeoxycholic acid-d_4_, glycocholic acid-d_4_, taurolithocholic acid-d_4_, taurochenodeoxycholic acid-d_4_, taurodeoxycholic acid-d_4_, tauroursodeoxycholic acid-d_4_, and taurocholic acid-d_4_.

### Short-chain fatty acid derivatization.

SCFAs were derivatized with 3-nitrophenylhydrazine (3-NPH) based on a method previously described by Borchers and coworkers (63). Fecal extracts (20 μl) were sequentially mixed with 10 μl of 200 mM 3-NPH–HCl in 50:50 acetonitrile-water (ACN-H_2_O) solution and 10 μl of 120 mM *N*-(3-dimethylaminopropyl)-*N*′-ethylcarbodiimide–HCl (EDC) in 50:44:6 ACN-H_2_O-pyridine solution. Mixtures were reacted at 40°C with 800 rpm mixing for 30 min. 3-NPH-derivatized samples were diluted with 960 μl of 50:50 ACN-H_2_O. One hundred microliters of 3-NPH-derivatized samples was mixed with 100 μl ^13^C_6_-3-NPH-derivatized SCFA internal standard containing derivatized acetic acid, propionic acid, isobutyric acid, butyric acid, 2-methyl butyric acid, isovaleric acid, and valeric acid.

### Liquid chromatography-mass spectrometry.

Relative quantitation of SCFAs and bile acids was performed by liquid chromatography-mass spectrometry (LC-MS) on an Agilent 1290 Infinity II LC coupled to a 6560 quadrupole time of flight (Q-TOF) instrument equipped with an Agilent Jet Stream (AJS) source operated in negative-ion mode. Chromatographic separation was performed using a Waters Acquity ultraperformance liquid chromatography (UPLC) BEH C_18_ column (1.7 μm, 2.1 by 100 mm). Mobile phases A and B were 0.01% formic acid in H_2_O and 0.01% formic acid in acetonitrile, respectively. SCFA analysis was performed with a flow rate of 0.3 ml·min^−1^, and the column temperature was maintained at 50°C. The initial solvent composition of 15% B was maintained for 2 min before increasing to 55% B at 11 min. The column was washed with 100% B for 3 min and equilibrated at initial solvent composition between injections for 5 min. MS spectra were recorded from *m/z* 50 to 1,000 at a rate of 1.5 spectra·s^−1^. Bile acid analysis was performed with a flow rate of 0.3 ml·min^−1^, and the column temperature was maintained at 50°C. The initial solvent composition of 5% B was maintained for 2.4 min before increasing to 30% B over 1 min and 70% B over 12 min. The column was washed with 100% B for 2.5 min and equilibrated at the initial solvent composition between injections for 4 min. MS spectra were recorded from *m/z* 50 to 1,200 at a rate of 1.5 spectra·s^−1^. The mass spectra for both methods were recalibrated to reference masses of *m/z* 112.9856 and 966.0007.

### LC-MS data analysis and metabolite quantitation.

The Agilent Profinder software was used for retention time alignment and feature extraction by batch recursive feature extraction method. Compound groups were annotated by their *m/z* and LC retention times compared to isotopically labeled internal standards, saved as Profinder Archive files, and exported with the integrated intensities from each sample. Relative quantitation was based on the integrated ion intensities of the features and internal standards. To test for the significance of the changes in the metabolite abundances, the ion intensities from each compound were log transformed and subtracted from the corresponding internal controls. The results (logR) were fitted to a mixed-effect model considering the interactions among the compounds, treatments, and time points. The between-subject effects and the dilution factors for each sample were modeled as “random” factors, while the mean-subtracted background level (logMedian.BK2) for each sample estimated from the median (logMedian.BK) of the unassigned features was incorporated as a covariate. The data are available at MetaboLights (study number MTBLS1257; https://www.ebi.ac.uk/metabolights/).

### Statistical analysis.

Microbiome analysis was performed in R. The majority of the analyses were done using the open-source package MGSAT, which wraps several R packages in order to perform -omics analyses (https://github.com/andreyto/mgsat). The scripts which generated the results presented in this paper are located in a directory, examples/16S/projects/mab_abx, versioned with Git tag mab_abx. Figures were generated with the R package ggplot2 (64). Richness and alpha- and beta-diversity metrics were calculated with the R package vegan (65) at the ASV level; all ASVs were included, regardless of abundance. To control for differences in sequencing depth per sample, samples were randomly rarefied to the minimum sample read count, and then each richness, alpha-diversity, or beta-diversity index was calculated. For each index, this process was repeated 400 times, and the results were averaged. Beta diversity was assessed using the Bray-Curtis dissimilarity index.

To estimate the statistical significance of the differences in compositional shifts caused by different treatments, we computed for each mouse the Bray-Curtis dissimilarity between day 7 and day 0 and used this within-mouse compositional shift in the *t* test for the difference in means between the pairs of treatment groups.

Richness and other abundance-based measures of alpha diversity in each sample were assessed using a series of Hill numbers (66), N*_q_*, where *q* was the order parameter ranging from zero to infinity. Hill numbers are a unified family of diversity indices (differing among themselves only by an exponent, *q*) that incorporate other measures of diversity and richness, expressing them on a uniform scale of the effective number of species (67). In particular, N_0_ is equivalent to richness, and N_1_ and N_2_ are equivalent to the exponentials of the Shannon index and the inverted Simpson index, respectively. Following the diversity analysis, sequence counts were normalized into simple proportions of taxa per sample to obtain the relative abundance profiles. For the genus-level analysis, genera from the lower quartiles of the mean relative abundance and mean incidence over all samples were discarded. The dynamic of the overall abundance profiles was plotted in the coordinates obtained from a correspondence analysis of a Bray-Curtis dissimilarity index computed between the geometric medians of samples in each treatment group at each time point.

### Data availability.

Data are available through the European Nucleotide Archive under accession number PRJEB34462.

## Supplementary Material

Supplemental file 1
