# Supplementary material for: Antibacterial Monoclonal Antibodies Do Not Disrupt the Intestinal Microbiome or Its Function
Source: Antimicrob Agents Chemother. 2020 Apr 21;64(5):e02347-19. doi: 10.1128/AAC.02347-19 (PMC7179586; doi:10.1128/AAC.02347-19)
Supplement: Supplemental file 1 [file AAC.02347-19-s0001.pdf]

Antibacterial Monoclonal Antibodies Do Not Disrupt the Intestinal Microbiome or its Function

Omari Jones-Nelson[1], Andrey Tovchigrechko[3], Matthew S. Glover[2], Fiona Fernandes[4], Udaya Rangaswamy[4], Liu Hui[4], David E. Tabor[4], Jonathan Boyd[1], Paul Warrener [1], Jose Martinez [1], Jamese J. Hilliard[1], C. Ken Stover[1], Wen Yu[3], Gina DAngelo[5], Sonja Hess[2], Taylor S. Cohen[1#], Bret R. Sellman[1#]

Department of Microbial Science[1], Antibody Discovery and Protein Expression[2], Research Bioinformatics[3], Translational Science[4], Statistical Sciences[5]

AstraZeneca, One MedImmune Way, Gaithersburg, MD 20878

Running Head: Anti-bacterial therapeutics influence the microbiome

Keywords: microbiome, antibiotics, monoclonal antibodies

Author Contributions: Conception and design: OJN, AT, PW, TSC, MSG, JJH, SH, BRS. Analysis and interpretation: OJN, MSG, AT, FF, UR, LH, DT, JB, WY, GD, SH, PW, CKS, TSC, BRS. Drafting the manuscript: OJN, TSC, BRS.

[#]Corresponding authors:
Taylor. S Cohen: taylor.cohen@astrazeneca.com
Bret R. Sellman: bret.sellman@astrazeneca.com
One Medimmune Way
Gaithersburg, MD 20877

*J.J.H. Is currently employed at Jansen Pharmaceuticals, Spring House PA

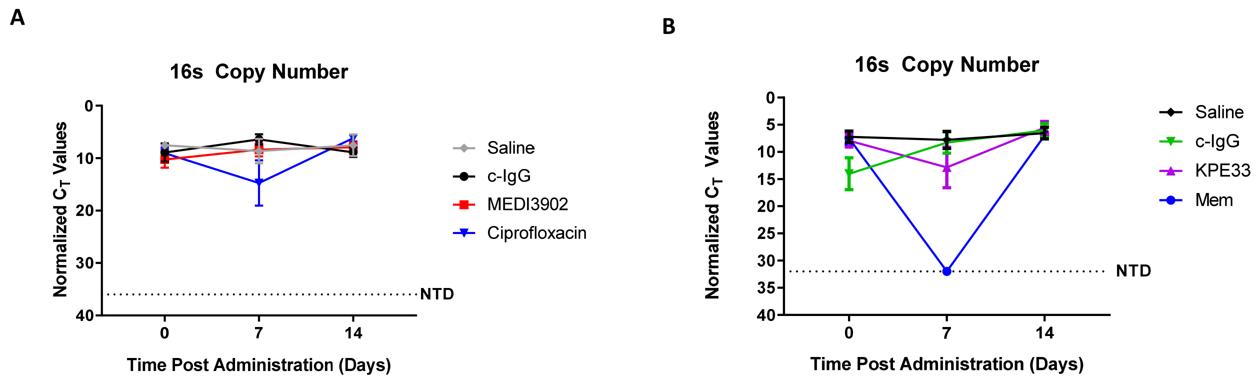

Supplemental Figure 1: Antibacterial mAbs do not change the microbial concentration of feces. The relative concentration of bacteria, as measured by 16s qPCR, in fecal samples of mice at day 0 (prior to treatment), 7 and 14 days post treatment of (A) saline, c-IgG, MEDI3902, or ciprofloxacin (B) saline, c-IgG, KPE33 or meropenem (MEM). All data are representative of at least two independent experiments, N = 5/group.

A

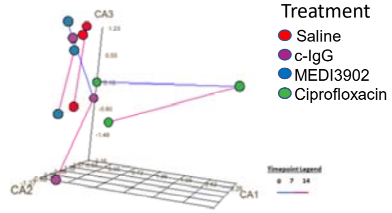

group.one	group.two	pvalue	adj. pvalue
MEDI3902	Saline	0.76527	2.29581
Cipro	Saline	0.00063	0.00188
c-IgG	Saline	0.89965	2.69896

B

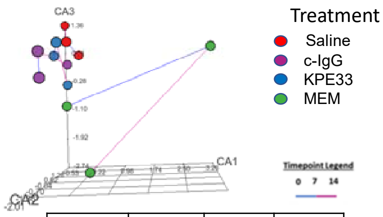

group.one	group.two	pvalue	adj. pvalue
KPE33	Saline	0.331391	0.994172
MEM	Saline	2.09E-07	6.26E-07
c-IgG	Saline	0.029162	0.087487

C

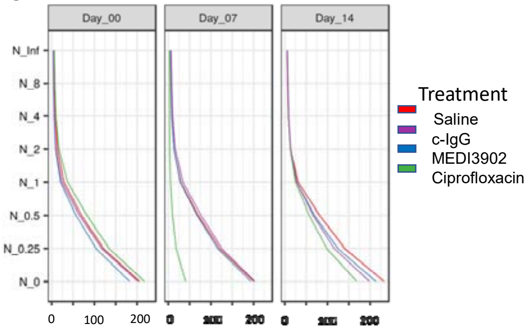

D

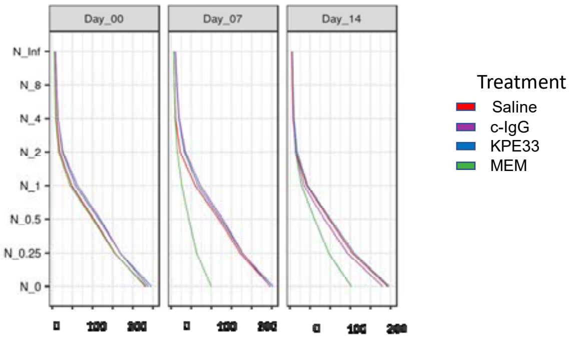

Supplemental Figure 2: Antibacterial mAbs do not change the overall microbial composition of feces. Correspondence Analysis (CA) plot of microbiota in fecal samples treated with (A) saline, c-IgG, MEDI3902 or ciprofloxacin (B) saline, c-IgG, KPE33 or meropenem (MEM). Each point represents the geometric median of the genus taxonomic profiles within each treatment group at days 0 (prior to treatment), 7, and 14. The timepoint legend indicates the period of collection with the blue line extending from time point 0 to 7, and the purple line extending from time point 7 to 14. (C,D) Plots of Abundance-based diversity indices (Hill numbers. Taxonomic profiles of individual observations were aggregated into geometric medians for each combination of treatment and time of sample collection. All data are representative of at least two independent experiments, N = 5/group.

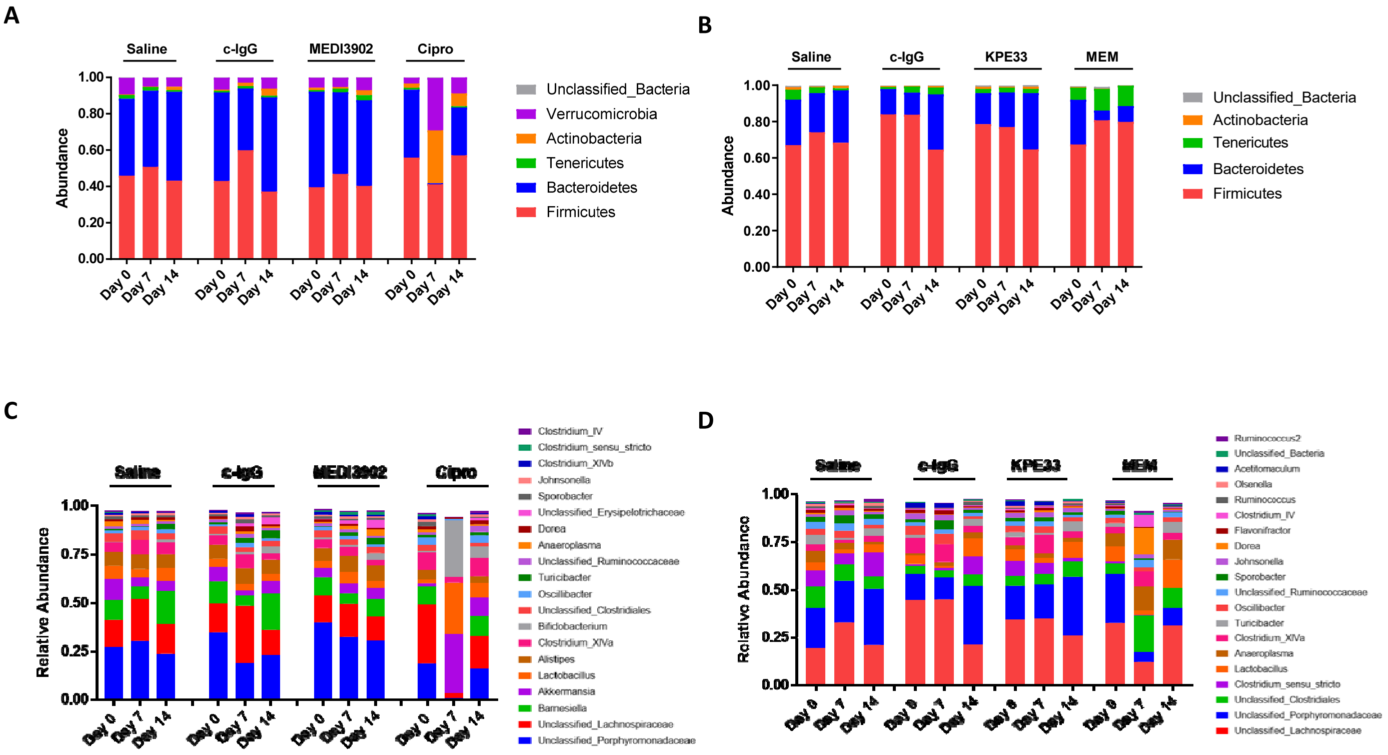

Supplemental Figure 3. Antibacterial mAbs do not change the bacterial abundance of feces. The relative abundance of major bacterial phyla of mice treated with (A) saline, c-IgG, MEDI3902 or ciprofloxacin (Cipro) (B) saline, c-IgG, KPE33 or meropenem (MEM). (C,D) The relative abundance of major bacterial genera of treated mice at day 0 (prior to treatment), 7 and 14. All data are representative of at least two independent experiments, N = 5/group.
